# Prognostic value of lymphocyte to monocyte ratio for the patients with bladder cancer: a systematic review and meta-analysis

**DOI:** 10.3389/fonc.2025.1601040

**Published:** 2025-10-03

**Authors:** Qiang Ren, Yumin Li, Hankai Chen, Yirun Chen

**Affiliations:** Department of Urology Surgery, The First People’s Hospital of Jiashan, Jiashan, Zhejiang, China

**Keywords:** lymphocyte, monocyte, LMR, bladder cancer, meta-analysis

## Abstract

**Objectives:**

To provide a meta-analysis evaluating the predictive value of lymphocyte to monocyte ratio (LMR) in the efficacy and prognosis of bladder cancer patients.

**Methods:**

Web of Science, Embase, Cochrane, and PubMed for literature searching up to November 2024 to identify research assessing the prognostic significance of LMR in bladder cancer patients. Outcomes included overall survival (OS), relapse-free survival (RFS), progression-free survival (PFS), and cancer-specific survival (CSS). Hazard ratios (HR) and 95% confidence intervals (CI) were used for data pooling of survival variables. In addition, for investigating potential heterogeneity sources and assessing the stability of the findings, sensitivity and subgroup analysis were performed. Review Manger 5.4 and STATA 15.1 were used to analyze.

**Results:**

Seventeen studies with 7,968 patients with bladder cancer included. The results indicated a notably shorter OS (HR: 1.56; 95% CI: 1.29, 1.89; *P* <0.00001), RFS (HR: 1.74; 95% CI: 1.27, 2.36; *P* = 0.0005), PFS (HR: 2.04; 95% CI: 1.58, 2.64; *P*<0.00001) and CSS (HR: 1.24; 95% CI: 1.01, 1.52; *P* = 0.04) in patients with low LMR compared to those with high LMR. Furthermore, subgroup analysis of OS found that study design, region, and age were the main factors affecting the correlation between LMR and OS.

**Conclusions:**

LMR can effectively predict the survival and recurrence risk of bladder cancer patients, helpingin the improvement of their prognosis. Future research should focus on large-scale, multicenter prospective cohort studies are still required in the future to evaluate the predictive value of LMR bladder cancer patients.

**Systematic Review Registration:**

https://www.crd.york.ac.uk/PROSPERO/view/CRD42024618066 PROSPERO (CRD42024618066).

## 1 Introduction

Bladder cancer is the most common cancer in the urinary system and poses a huge threat to human health. In 2018, an estimated 549,393 people were diagnosed with bladder cancer for the first time worldwide, with a direct death toll of 199,922.

In 2020, around 81,400 cases of bladder cancer were newly identified in the United States, with a death toll of 17,980 (1, 2). The regions with the highest incidence of bladder cancer are North America, Europe, and West Asia, respectively, but the mortality rate in developing regions is higher than that in developed regions (3). The cause of bladder cancer is relatively complex and unclear, including both genetic factors and external environmental factors (4, 5). Some reports believe that it is related to long-term smoking, abnormal tryptophan metabolism in the body, and long-term exposure to aromatic chemicals (5). Symptoms include hematuria, lower abdominal mass, difficulty urinating, bladder irritation, etc. (6, 7), which seriously affect people’s physical condition and gradually attract the attention of the medical community.

Advancements in minimally invasive surgical techniques have led to transurethral resection of bladder tumor (TURBT) being increasingly recognized as the primary approach for managing non-muscle invasive bladder cancer (NMIBC), while also serving as an essential diagnostic method. Generally, the pathological grade and stage are determined by the pathological results after TURBT (4, 8). Among patients with newly diagnosed bladder cancer, 75% are NMIBC. Among NMIBC, high-risk bladder urothelial carcinoma has higher invasiveness and diverse disease progression, and the postoperative recurrence rate can reach 60% to 70% (6, 7). The high recurrence rate of bladder cancer is associated with a variety of factors (9, 10). Therefore, exploring the relationship between various factors and the prognosis of bladder cancer can provide guidance for the clinical prevention and treatment and prognosis evaluation of this condition.

The relationship between tumors and inflammation has gradually become a hot topic of research. Studies have shown that tumor-related inflammation may promote tumor invasion, infiltration and metastasis (11–14). The lymphocyte to monocyte ratio (LMR) is a simple, rapid and economic indicator that can simultaneously reflect the status of lymphocytes and monocytes. It can reflect the dynamic relationship between the body’s anti-tumor immunity and inflammatory response to a certain extent, and has the predictive advantages of both lymphocytes and monocytes for tumor prognosis. At present, research has indicated that preoperative LMR is related to the prognosis of various malignancies, including ovarian cancer, breast cancer, colon cancer, lung cancer, and others (15–18). However, its relationship with the postoperative prognosis of bladder cancer patients has not yet been clarified.

Although Ma et al. (19) conducted a meta-analysis in 2019 assessing the predictive value of LMR for the prognosis of individuals with bladder cancer, numerous new clinical studies have emerged since its publication to examine the prognostic value of LMR for bladder cancer, and the conclusions are inconsistent. Therefore, this article aims to use LMR, an easily detectable peripheral circulating marker, as the research object, by conducting meta-analysis. Building on the existing meta-analysis, combined with the latest research data, the goal is to further evaluate the prognostic value of LMR of individuals diagnosed with bladder cancer and provide the latest evidence for the creation of a bladder cancer prognosis prediction model.

## 2 Methods

### 2.1 Literature search

This analysis was conducted following the PRISMA (Preferred Reporting Items for Systematic Reviews and Meta-Analysis) 2020 statement (20) and was registered in the PROSPERO database (CRD42024618066). PubMed, Embase, Web of Science, and Cochrane databases were used for literature searching up to November, 2024 for studies assessing the prognostic value of LMR in individuals diagnosed with bladder cancer. We conducted the literature search using the terms: “lymphocyte”, “monocyte”, “LMR”, “urinary bladder neoplasms”, and “bladder cancer”, etc. The specific search strategies used in PubMed are: ((((“Lymphocytes”[Mesh]) OR (((Lymphocyte) OR (Lymphoid Cells)) OR (Lymphoid Cell))) AND ((“Monocytes”[Mesh]) OR (Monocyte))) AND (ratio)) AND ((“Urinary Bladder Neoplasms”[Mesh]) OR ((((((((((Urinary Bladder Neoplasm) OR (Bladder Neoplasms)) OR (Bladder Neoplasm)) OR (Bladder Tumors)) OR (Bladder Tumor)) OR (Urinary Bladder Cancer)) OR (Bladder Cancer)) OR (Bladder Cancers)) OR (Cancer of Bladder)) OR (Cancer of the Bladder))). Next, we conducted a manual review of the reference lists of every study that was considered. Two authors conducted the retrieval and assessment of qualifying articles independently. Any disagreements in the literature retrieval process were resolved through discussion. **Supplementary Table S1** contains the comprehensive literature search strategy.

### 2.2 Inclusion and exclusion criteria

Inclusion criteria for articles were determined based on the following criteria: (1) the study design had to be a randomized controlled trial, cohort study, or case-control study; (2) the study was performed on patients with bladder cancer; (3) studies assessed the role of LMR in the prognosis of bladder cancer patients; (4) one or more survival outcomes (such as: overall survival (OS), disease-free survival (DFS), relapse-free survival (RFS), progression-free survival (PFS), cancer-specific survival (CSS), etc.) were assessed; (5) sufficient data to analyze hazard ratio (HR), risk ratio (RR) or odds ratio (OR). We excluded non-original studies (such as abstracts, letters, corrections, comments, and replies), study protocols, unpublished research, reviews, and studies that lacked sufficient data.

### 2.3 Data abstraction

Two authors independently performed data extraction, and any discrepancies were resolved by another author. The following data was extracted from the included studies: first author name, year of publishing, study duration, study country, study design, types of tumors, treatments, sample size, age, LMR cut-off, OS, CSS, PFS and RFS. In cases where the available data were incomplete, the primary authors were approached to provide the full data, if accessible.

### 2.4 Quality evaluation

The quality of the selected cohort studies was evaluated according to the Newcastle-Ottawa Scale (NOS) (21), with studies scoring between 7 and 9 points classified as high quality (22).

The evaluation of all selected studies was independently conducted by two authors, with any discrepancies were resolved via discussion.

### 2.5 Statistical analysis

Review Manager 5.4.1 was used for analyzing. Survival data were synthesized using hazard ratios (HR) and 95% confidence intervals (CIs). Heterogeneity for each outcome was evaluated through inconsistency index (*I* ^2^) and the chi-squared (χ^2^) test (Cochran’s *Q*) (23). High heterogeneity was defined as a χ^2^ *P* value below 0.1 or an *I* ^2^ value exceeding 50%.

The random-effects model was used to estimate the overall HR for all outcomes. Additionally, we conducted subgroup analyses to assess potential confounders, provided that sufficient data were available. Sensitivity analysis was also employed to determine each study’s influence on the overall HR for each outcome. Additionally, we evaluated the publication bias by funnel plots and Egger’s regression tests (24) conducted with Stata 15.1 edition (Stata Corp, College Station, Texas, USA). Publication bias was considered statistically significant if the P-value was <0.05.

## 3 Results

### 3.1 Literature retrieval, study characteristics, and baseline

**Figure 1:**
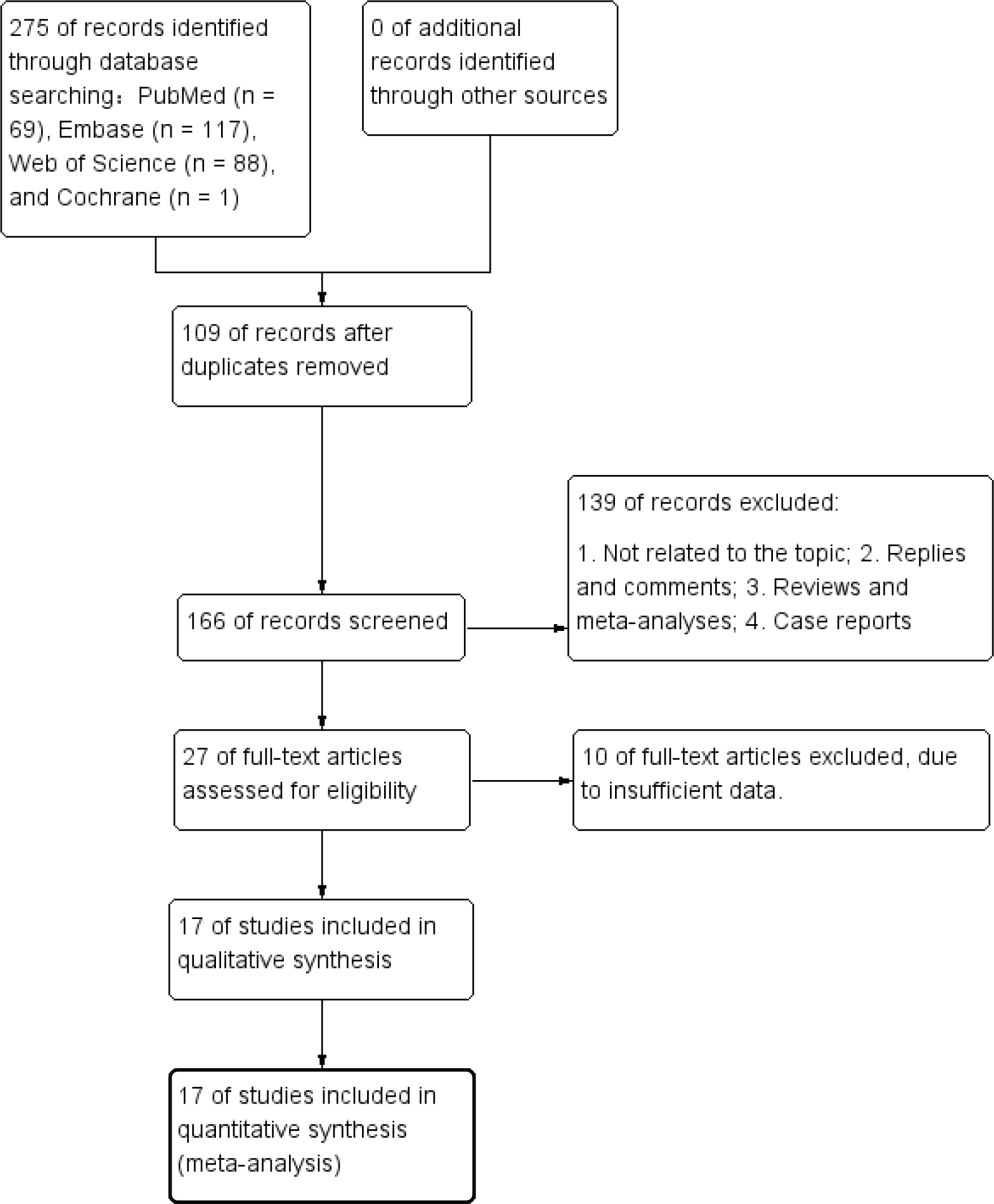
Flowchart of the systematic search and selection process.

**Figure 1** presents the diagram outlining the process of literature retrieval and selection. A comprehensive literature search identified a total of 275 relevant studies across four databases: PubMed (n = 69), Embase (n = 117), Web of Science (n = 88), and Cochrane (n = 1). After eliminating duplicates, 166 records underwent screening based on their titles and abstracts. Finally, 17 cohort studies including 7,968 patients were incorporated into the meta-analysis (25–41). The characteristics and quality evaluation are detailed in **Table 1**.

**Table 1:** Characteristics and quality assessment of included cohort studies.

Study	Study period	Country	Study design	Types of tumors	Treatments	No. of patients	Gender		Mean/median age	LMR cut-off	NOS
							Male	Female			
Adamkiewicz 2021 (25)	NA	Poland	Retrospective cohort	NMIBC	TURBT followed by BCG immunotherapy	125	107	18	69.4	3.25	7
Albisinni 2019 (26)	2013-2018	Belgium	Prospective cohort	MIBC	Radical cystectomy	134	110	24	72	1.69	7
Batur 2021 (27)	2009-2019	Turkey	Retrospective cohort	NMIBC and MIBC	TURBT	138	123	15	68.05	NA	7
Cantiello 2018 (28)	2002-2012	Italy	Prospective cohort	NMIBC	TURBT	1155	957	198	71	3.41	7
D’Andrea 2017 (29)	2006-2016	Japan	Retrospective cohort	NMIBC and MIBC	Radical cystectomy	4198	3362	836	67	3.5	8
Karan 2023 (30)	2008-2020	Turkey	Retrospective cohort	MIBC	Chemotherapy with or without definitive/palliative radiotherapy	214	198	16	67	2.73	7
Kim 2021 (31)	2000-2007	Korea	Retrospective cohort	NMIBC	TURBT	151	127	24	63.6	NA	8
Kool 2022 (32)	2001-2017	Canada	Retrospective cohort	MIBC	Initial maximal transurethral resection of the bladder tumor, followed by radiotherapy with concurrent chemotherapy	176	134	42	75	NA	7
Lee 2015 (33)	2011-2013	UK	Retrospective cohort	NMIBC and MIBC	TURBT	226	174	52	NA	1.8	8
Li 2022 (34)	2014-2020	China	Retrospective cohort	NMIBC	TURBT followed by BCG immunotherapy	197	170	27	64.27	3.6	8
Shi 2020 (35)	2009-2018	China	Retrospective cohort	MIBC	Radical cystectomy	203	176	27	66	1.85	7
Tang 2020 (36)	2014-2020	China	Retrospective cohort	NMIBC and MIBC	Radical cystectomy	79	70	9	63.6	2.8	7
Wang 2019 (37)	2010-2018	China	Retrospective cohort	NMIBC	TURBT	358	291	67	68.6	2.8	8
Yildiz 2021 (38)	2017-2018	Turkey	Prospective cohort	NMIBC	TURBT	94	85	9	NA	3.63	7
Yoshida 2016 (39)	1995-2013	Japan	Retrospective cohort	NMIBC and MIBC	Radical cystectomy	302	238	64	NA	3.41	7
Zhang 2015 (40)	2009	China	Retrospective cohort	NMIBC and MIBC	Radical cystectomy	124	100	24	NA	4	9
Zhang 2024 (41)	2012-2022	China	Retrospective cohort	MIBC	Radical cystectomy	94	84	10	71.6	2.98	8

### 3.2 OS

**Figure 2:**
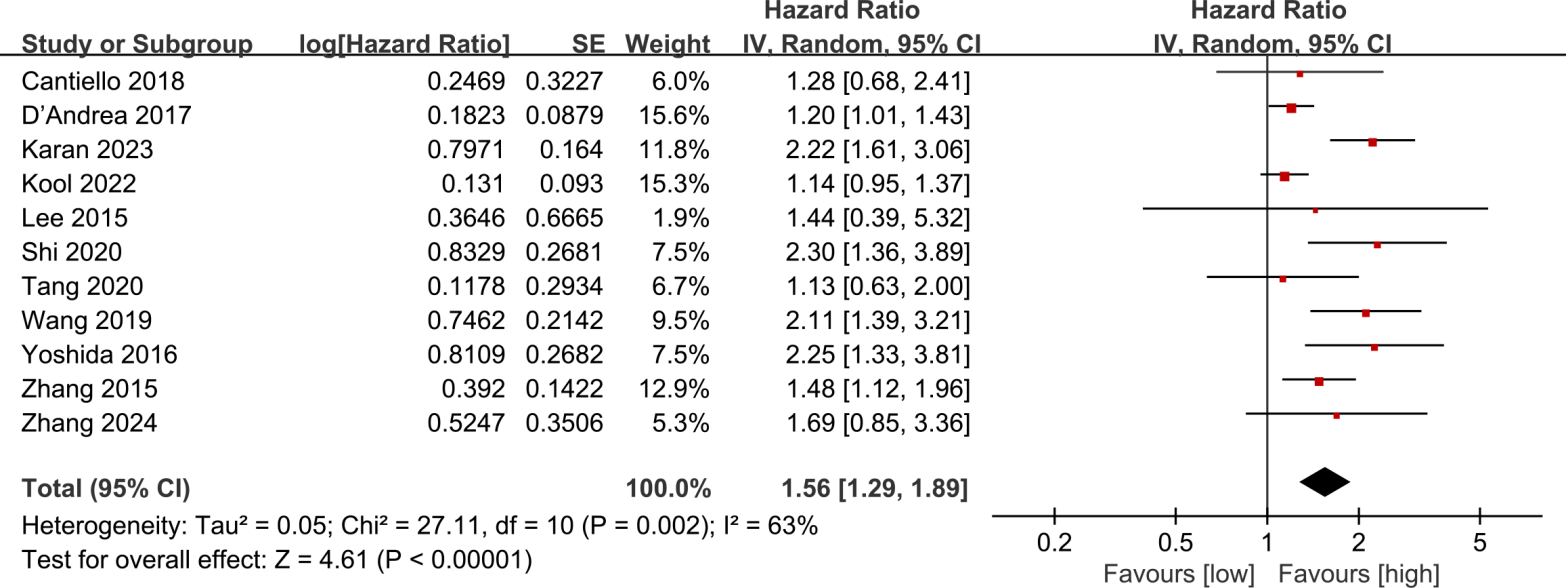
Forest plots of OS.

Data on OS were pooled from 11 cohort studies, with the meta-analysis indicating a significantly shorter OS in the low LMR group (HR: 1.56; 95% CI: 1.29, 1.89; *P* <0.00001). Significant heterogeneity was detected (*I* ^2^ = 63%, *P* = 0.002) (**Figure 2**). Subgroup analysis was performed according to study design, types of tumors, region, sample size, age, and cut-off. The results showed that LMR had no significant predictive value for OS in the prospective study subgroup, European and American subgroups, and subgroups aged ≥ 70 years. In the remaining subgroups, LMR was still significantly associated with OS. The detailed subgroup analysis results are shown in **Table 2**.

**Table 2:** Subgroup analysis of LMR and OS.

Subgroup	OS			
	Study	HR [95%CI]	*P* value	*I* ^2^
Total	11	1.56 [1.29-1.89]	<0.00001	63%
Study design				
Prospective	1	1.28 [0.68-2.41]	0.44	/
Retrospective	10	1.58 [1.30-1.94]	<0.00001	67%
Types of tumors				
NMIBC	2	1.74 [1.08-2.80]	0.02	40%
MIBC	4	1.73 [1.11-2.68]	0.01	82%
Region				
Asia	8	1.70 [1.35-2.14]	<0.00001	66%
Europe	2	1.31 [0.74-2.31]	0.35	0%
America	1	1.14 [0.95-1.37]	0.16	/
Sample size				
≥200	7	1.78 [1.33-2.40]	0.0001	70%
<200	4	1.25 [1.07-1.47]	0.005	8%
Mean/median age				
≥70y	3	1.18 [0.99-1.40]	0.06	0%
<70	5	1.70 [1.20-2.40]	0.003	78%
LMR cut-off				
≥3	4	1.42 [1.12-1.79]	0.004	48%
<3	6	1.96 [1.60-2.40]	<0.00001	0%

### 3.3 RFS

**Figure 3:**
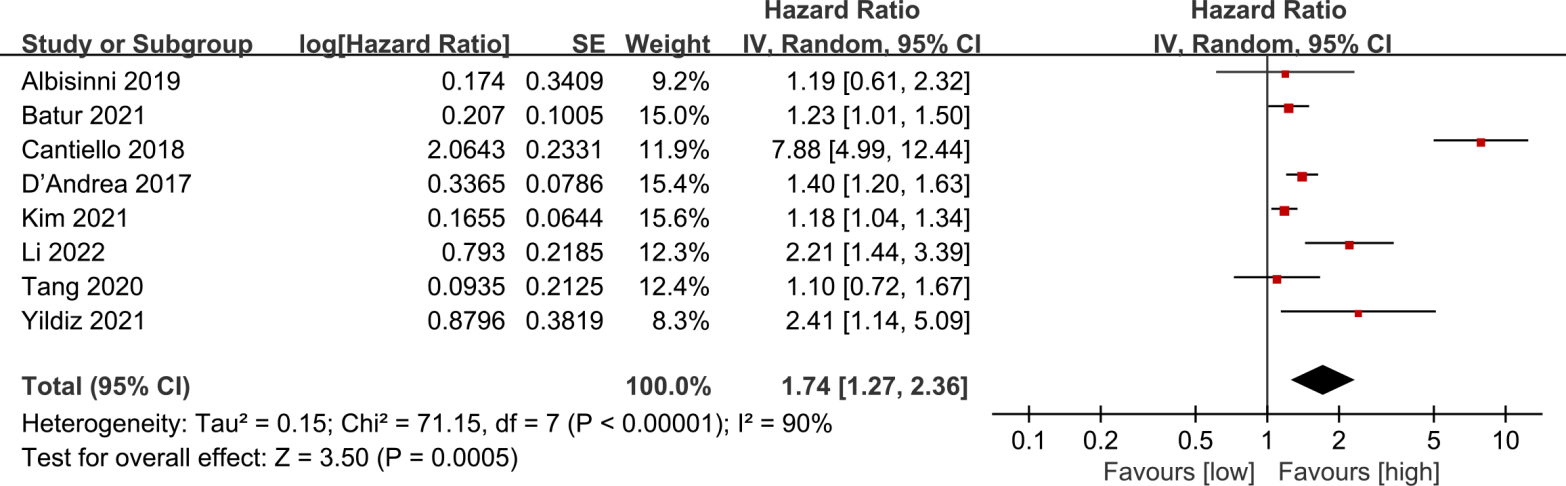
Forest plots of RFS.

Data on RFS were aggregated from 8 cohort studies, with meta-analysis demonstrating a notably reduced in the low LMR group (HR: 1.74; 95% CI: 1.27, 2.36; *P* = 0.0005). Significant heterogeneity was detected (*I* ^2^ = 90%, *P* <0.00001) (**Figure 3**).

### 3.4 PFS

**Figure 4:**
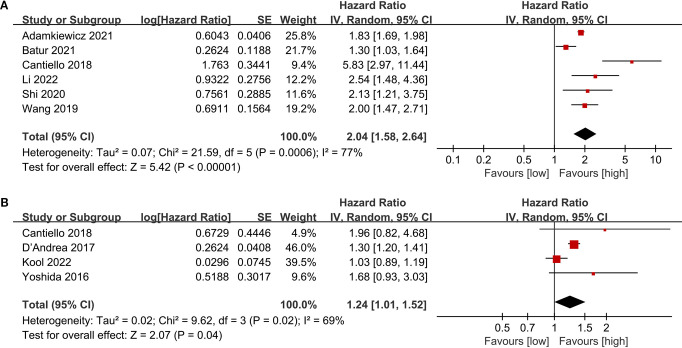
Forest plots of PFS **(A)**, and CSS **(B)**.

The PFS data were combined from 6 cohort studies, with the meta-analysis showing a significantly reduced PFS in the low LMR group (HR: 2.04; 95% CI: 1.58, 2.64; *P*<0.00001). Significant heterogeneity was detected (*I* ^2^ = 70%, *P* = 0.0006) (**Figure 4A**).

### 3.5 CSS

The CSS data were combined from 4 cohort studies, and the meta-analysis indicated a considerably shorter CSS in the low LMR group (HR: 1.24; 95% CI: 1.01, 1.52; *P* = 0.04). Notable heterogeneity was detected (*I* ^2^ = 69%, *P* = 0.02) (**Figure 4B**).

### 3.6 Publication bias and sensitivity analysis

**Figure 5:**
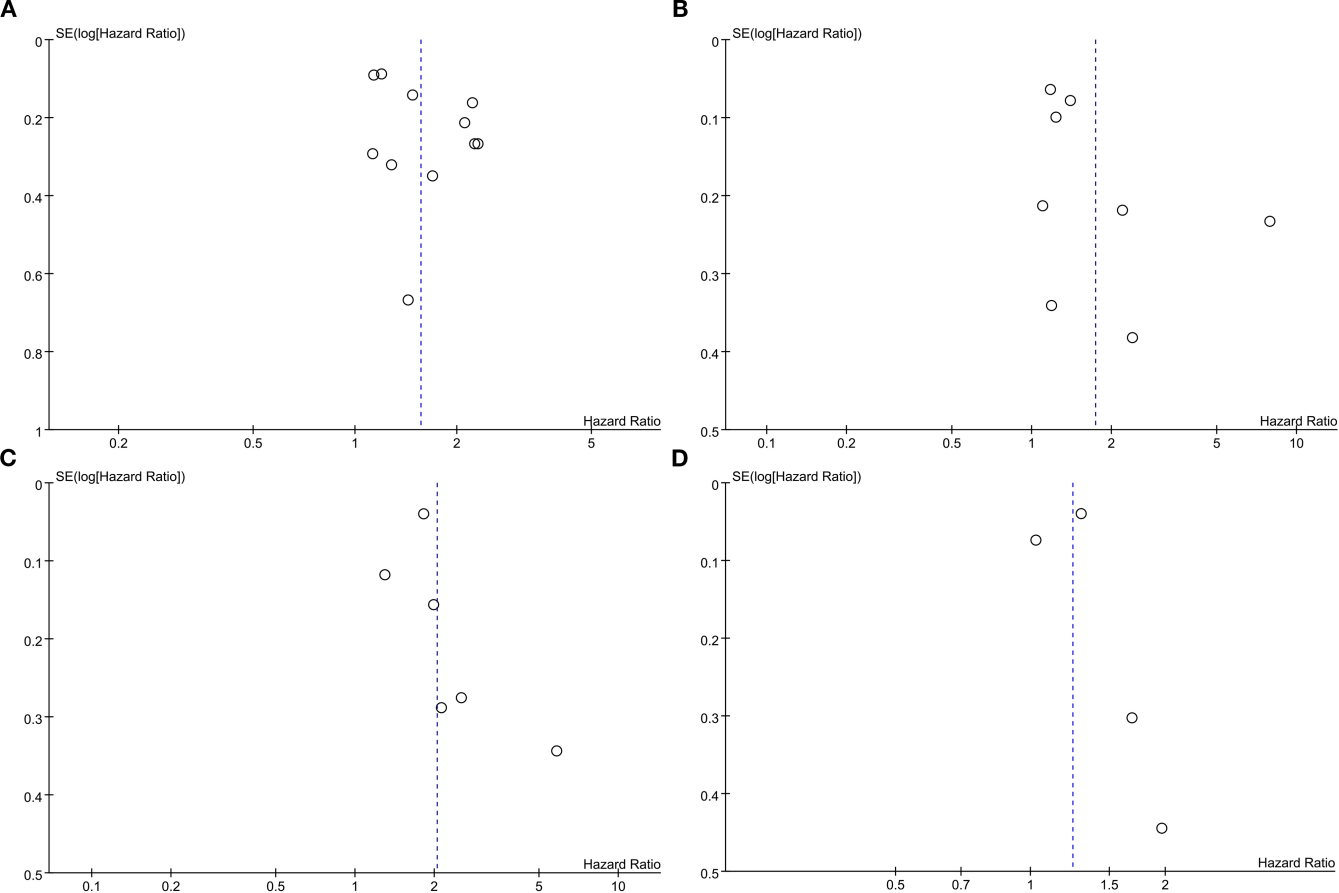
Funnel plots of OS **(A)**, RFS **(B)**, PFS **(C)** and CSS **(D)**.

**Figure 6:**
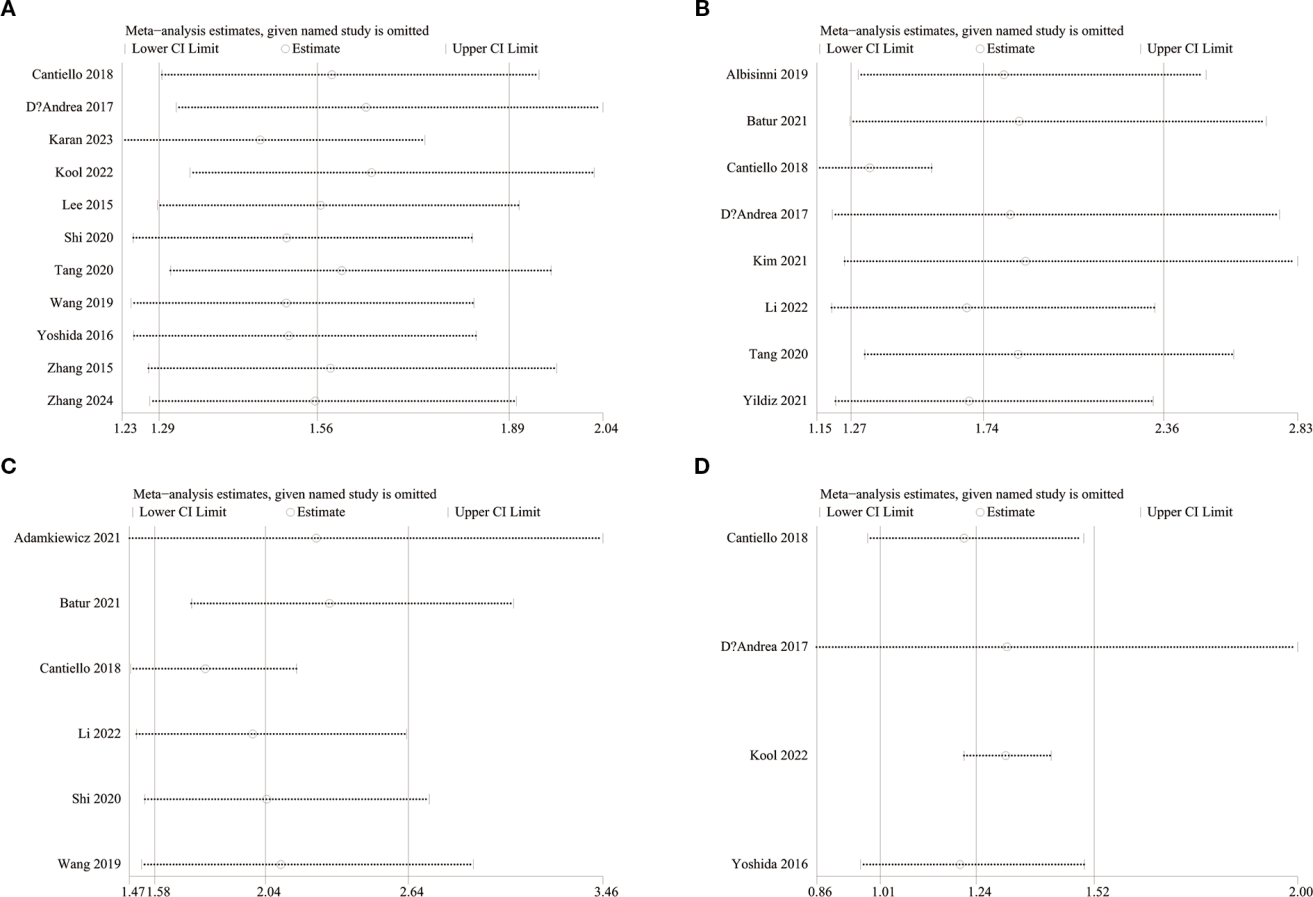
Sensitivity analysis of OS **(A)**, RFS **(B)**, PFS **(C)** and CSS **(D)**.

To evaluate potential publication bias, funnel plots and Egger’s regression tests were performed for OS, RFS, PFS, and CSS. No indication of publication bias was detected for OS (Egger’s test *P* = 0.080) (**Figure 5A**), RFS (Egger’s test *P* = 0.169) (**Figure 5B**), PFS (Egger’s test *P* = 0.434) (**Figure 5C**), and CSS (Egger’s test *P* = 0.830) (**Figure 5D**), either statistically (via Egger’s test) or visually (via funnel plots). Additionally, we conducted sensitivity analysis on the OS, RFS, PFS, and CSS to evaluate the impact of each cohort study on the overall HR by sequentially excluding one study at a time. Sensitivity analysis showed that excluding each cohort study did not notably affect the total HR for OS (**Figure 6A**), RFS (**Figure 6B**) and PFS (**Figure 6C**). However, the sensitivity analysis of CSS found that when the data of Cantiello 2018, D’Andrea 2017, and Yoshida 2016 were excluded, the correlation between LMR and CSS changed from significant to insignificant, suggesting that this indicator is currently unstable (**Figure 6D**).

## 4 Discussion

In order to more effectively assess the prognostic risk and achieve the goal of personalized precision treatment, prognostic markers with high predictive value are needed. Conventional peripheral blood testing has the advantages of being easy to operate and inexpensive. Bladder cancer is a heterogeneous disease that is prone to recurrence and invasion, and has a high mortality rate. Exploring peripheral blood markers with predictive value can assist in identifying high-risk groups as early as possible so that active intervention can be taken to enhance the prognosis of individuals diagnosed with a bladder cancer. This study identified 17 clinical studies through systematic literature retrieval and screening and conducted a meta-analysis to explore the predictive significance of LMR on the prognostic risk of bladder cancer patients and its potential influencing factors.

This study found that LMR can significantly predict OS, RFS, PFS, and CSS in bladder cancer. Except for the sensitivity analysis of CSS, which revealed instability, the significant correlations between LMR and OS, RFS, and PFS were stable, and no significant publication bias was observed, which further demonstrated the reliability of the evidence in this study. Consistent with this study, a meta-analysis by Ma et al. (19) summarized 9 studies and revealed a strong association between LMR and OS, RFS, and CSS in patients with bladder cancer. However, unlike this study, the study by Ma et al. did not report the link between LMR and PFS due to insufficient data. Therefore, this study made up for the shortcomings of previous studies on the basis of further confirming the predictive value of LMR for individuals diagnosed with bladder cancer.

Regarding the findings of subgroup analysis, this study found that study design, region, and age were the main factors affecting the correlation between LMR and OS. First, the prospective study did not observe a correlation between LMR and OS, but considering that only one study was prospectively designed, the conclusion lacks reliability and needs further research to confirm. Second, most of the literature incorporated in this study came from Asia, and there were only three literatures from Europe and America, so the negative results may also be related to insufficient sample size. However, the differences in bladder cancer incidence and prognosis between Asia and Europe and the United States may also affect the predictive value of LMR, but further research is needed to confirm. Finally, this study demonstrated that the predictive value of LMR for OS is better in individuals under 70 years old, and patients ≥70 years old are not sensitive to LMR. In 2021, the European Association of Urology (EAU) published a new NMIBC prognostic factor risk group study. The study received data information from 5295 patients from 17 institutions, and finally the data of 3401 newly diagnosed patients were used to estimate the study. The statistical results included high age (>70 years old) as a prognostic risk factor for NMIBC (42). Considering the significant changes in the immune and inflammatory levels of elderly patients, we speculate that the loss of predictive value of LMR in patients aged ≥70 years may be because its predictive efficacy is masked by more significant risk factors associated with aging. This also suggests that the impact of age should be considered when using LMR to stratify patients clinically.

LMR represents the ratio of lymphocyte count to monocyte count. Lymphocytes play a crucial role in inducing cytotoxic cell death and in preventing tumor cell proliferation and metastasis (43–45). Lymphocytopenia often reflects the severity of the disease and may lead to tumor cells evading immune surveillance by tumor-infiltrating lymphocytes (TIL) (46, 47). TIL consists of lymphocytes which migrate to the tumor microenvironment (48). TIL can recognize tumor cells and initiate cytotoxic induction of apoptotic cell death (49). Low levels of TIL have been identified as a predictor of poor outcomes in patients with colorectal cancer (50, 51). In addition, studies have found that monocytes can promote tumor progression and metastasis, with several proinflammatory cytokines produced by monocytes, such as TNF-α and IL-1, being linked to poor prognosis in cancer patients (52, 53). Tumor-associated macrophages also originate from circulating monocytes and have an immunosuppressive effect (54, 55). Therefore, a reduction in LMR may be related with a poor prognosis in individuals diagnosed with cancer. Kawai et al. reported that low LMR can effectively predict poor survival in pancreatic cancer and speculated that lymphocytes, mainly TILs, play a vital role in enhancing antitumor immune responses (56). CD4+ T lymphocytes are crucial for initiating and maintaining antitumor immunity through cytokine secretion, including IL-2, or by activating antigen-presenting cells. CD8+ T lymphocytes detect tumor-associated antigens, directly attack tumor cells, and prevent their proliferation (45). Monocytes, in contrast, contribute to tumor invasion and support angiogenesis. Tumor-associated macrophages, derived from circulating monocytes, inhibit T cell-mediated antitumor immunity and secrete powerful pro-angiogenic factors among which are vascular endothelial growth factor (VEGF)-A and TNF-α (53, 57). Increased monocyte counts and decreased lymphocyte counts promote the formation of a tumor inflammatory microenvironment (56). Another study indicated that elevated levels of tumor-infiltrating CD4+ and CD8+ T lymphocytes within the tumor microenvironment were strongly linked to prolonged overall survival (OS) (58).

However, certain limitations of this meta-analysis should be acknowledged. Firstly, due to the fundamental characteristics of clinical research, retrospective cohort studies constitute most of the research analyzed in this study. It is widely acknowledged that potential confounding factors and risk of bias are the biggest disadvantages of retrospective studies. In addition, most of the studies analyzed originate from Asia, while a smaller number come from Europe and America, and there is a notable absence of data from Africa and Oceania. As a result, it remains uncertain whether the results of this study can be generalized to other countries. Finally, there is obvious instability in the results of LMR in predicting CSS, so caution should be used when interpreting the predictive value of LMR for CSS. Subsequent studies should focus on collecting more comprehensive individual patient data (IPD) to enable in-depth, multivariate model-based meta-analysis of IPDs based on uniform adjustment for multiple potential confounders (including detailed comorbidities, inflammatory status, other blood parameters, and tumor characteristics). This will greatly enhance the assessment of the independent predictive value of LMR and help develop more accurate clinical prediction tools. While acknowledging some inherent limitations, this analysis is the most recent and largest one reporting the prognostic predictive value of LMR in individuals diagnosed with this disease. This study highlights the critical role of assessing LMR levels in the clinical treatment. Constructing a highly efficient bladder cancer risk prediction model incorporating inflammatory indicators such as LMR could enhance patient outcomes and quality of life.

## 5 Conclusion

As a clinically accessible, inexpensive, and non-invasive biomarker, LMR can effectively predict the survival and recurrence risk of individuals diagnosed with bladder cancer and help improve their prognosis. Considering the limitations of this article, such as the majority of retrospective studies, regional selection bias, potential instability, and heterogeneity, further large-scale, multicenter, prospective cohort studies are still required in the future to evaluate the prognostic value of LMR for patients.

## Data Availability

The original contributions presented in the study are included in the article/**Supplementary Material**. Further inquiries can be directed to the corresponding author.

## References

[1] Siegel RL Miller KD Wagle NS Jemal A . Cancer statistics, 2023. CA: Cancer J Clin. (2023) 73:17–48. doi: 10.3322/caac.21763, PMID: 36633525

[2] Bray F Ferlay J Soerjomataram I Siegel RL Torre LA Jemal A . Global cancer statistics 2018: GLOBOCAN estimates of incidence and mortality worldwide for 36 cancers in 185 countries. CA: Cancer J Clin. (2018) 68:394–424. doi: 10.3322/caac.21492, PMID: 30207593

[3] Ferlay J Soerjomataram I Dikshit R Eser S Mathers C Rebelo M . Cancer incidence and mortality worldwide: sources, methods and major patterns in GLOBOCAN 2012. Int J cancer. (2015) 136:E359–E86. doi: 10.1002/ijc.v136.5 25220842

[4] Heard JR Ahdoot M Theodorescu D Mitra AP . Biomarkers of treatment response in bladder cancer. Expert Rev Mol diagnostics. (2024), 1–13. doi: 10.1080/14737159.2024.2428747, PMID: 39535158

[5] Chen C Fan G Li P Yang E Jing S Shi Y . Effect of smoking on the recurrence and progression of non-muscle-invasive bladder cancer. Clin Trans Oncol Off Publ Fed Spanish Oncol Societies Natl Cancer Institute Mexico. (2024). doi: 10.1007/s12094-024-03694-z, PMID: 39266874

[6] Yang T Luo W Yu J Zhang H Hu M Tian J . Bladder cancer immune-related markers: diagnosis, surveillance, and prognosis. Front Immunol. (2024) 15:1481296. doi: 10.3389/fimmu.2024.1481296, PMID: 39559360 PMC11570592

[7] Ma X Zhang Q He L Liu X Xiao Y Hu J . Artificial intelligence application in the diagnosis and treatment of bladder cancer: advance, challenges, and opportunities. Front Oncol. (2024) 14:1487676. doi: 10.3389/fonc.2024.1487676, PMID: 39575423 PMC11578829

[8] D’Andrea D Mostafid H Gontero P Shariat S Kamat A Masson-Lecomte A . Unmet need in non-muscle-invasive bladder cancer failing bacillus calmette-guérin therapy: A systematic review and cost-effectiveness analyses from the international bladder cancer group. Eur Urol Oncol. (2024)., PMID: 39550339 10.1016/j.euo.2024.10.012

[9] Lv M Shang S Liu K Wang Y Xu P Song H . Revitalizing bacillus calmette-guérin immunotherapy for bladder cancer: nanotechnology and bioengineering approaches. Pharmaceutics. (2024) 16. doi: 10.3390/pharmaceutics16081067, PMID: 39204412 PMC11359013

[10] Abou Chakra M Luo Y Duquesne I A O'Donnell M . Update on the mechanism of action of intravesical BCG therapy to treat non-muscle-invasive bladder cancer. Front bioscience (Landmark edition). (2024) 29:295. doi: 10.31083/j.fbl2908295, PMID: 39206898

[11] Tezcan G Yakar N Hasturk H Van Dyke TE Kantarci A . Resolution of chronic inflammation and cancer. Periodontology 2000. (2024) 96:229–49. doi: 10.1111/prd.12603, PMID: 39177291

[12] Shen G Wang Q Li Z Xie J Han X Wei Z . Bridging chronic inflammation and digestive cancer: the critical role of innate lymphoid cells in tumor microenvironments. Int J Biol Sci. (2024) 20:4799–818. doi: 10.7150/ijbs.96338, PMID: 39309440 PMC11414386

[13] Justus CR Marie MA Sanderlin EJ Yang LV . The roles of proton-sensing G-protein-coupled receptors in inflammation and cancer. Genes. (2024) 15. doi: 10.3390/genes15091151, PMID: 39336742 PMC11431078

[14] Bick F Blanchetot C Lambrecht BN Schuijs MJ . A reappraisal of IL-9 in inflammation and cancer. Mucosal Immunol. (2024). doi: 10.1016/j.mucimm.2024.10.003, PMID: 39389468

[15] Zhang YY Liu FH Wang YL Liu JX Wu L Qin Y . Associations between peripheral whole blood cell counts derived indexes and cancer prognosis: An umbrella review of meta-analyses of cohort studies. Crit Rev oncology/hematology. (2024) 204:104525. doi: 10.1016/j.critrevonc.2024.104525, PMID: 39370059

[16] Pang H Dai L Chen L Chen X Chen Z Zhang S . Prognostic value of the advanced lung cancer inflammation index in patients with gastric cancer after radical gastrectomy: a propensity-score matching cohort study and meta-analysis. BMC cancer. (2024) 24:583. doi: 10.1186/s12885-024-12349-9, PMID: 38741082 PMC11089784

[17] Portale G Bartolotta P Azzolina D Gregori D Fiscon V . Prognostic role of platelet-to-lymphocyte ratio, neutrophil-to-lymphocyte, and lymphocyte-to-monocyte ratio in operated rectal cancer patients: systematic review and meta-analysis. Langenbeck’s Arch Surg. (2023) 408:85. doi: 10.1007/s00423-023-02786-8, PMID: 36781510

[18] Savioli F Morrow ES Dolan RD Romics L Lannigan A Edwards J . Prognostic role of preoperative circulating systemic inflammatory response markers in primary breast cancer: meta-analysis. Br J Surg. (2022) 109:1206–15. doi: 10.1093/bjs/znac319, PMID: 36130112

[19] Ma JY Hu G Liu Q . Prognostic significance of the lymphocyte-to-monocyte ratio in bladder cancer undergoing radical cystectomy: A meta-analysis of 5638 individuals. Dis markers. (2019) 2019:7593560. doi: 10.1155/2019/7593560, PMID: 31089397 PMC6476040

[20] Page MJ McKenzie JE Bossuyt PM Boutron I Hoffmann TC Mulrow CD . The PRISMA 2020 statement: an updated guideline for reporting systematic reviews. BMJ (Clinical Res ed). (2021) 372:n71. 10.1136/bmj.n71 PMC8005924 33782057

[21] Wells G Shea B O’Connell D Peterson J Welch V Losos M . The Newcastle-Ottawa Scale (NOS) for assessing the quality of nonrandomised studies in meta-analyses 2011 . Available online at: http://www.ohri.ca/programs/clinical_epidemiology/oxford.asp (Accessed January 2025).

[22] Kim SR Kim K Lee SA Kwon SO Lee JK Keum N . Effect of red, processed, and white meat consumption on the risk of gastric cancer: an overall and dose-Response meta-analysis. Nutrients. (2019) 11., PMID: 30979076 10.3390/nu11040826 PMC6520977

[23] Higgins JP Thompson SG . Quantifying heterogeneity in a meta-analysis. Stat Med. (2002) 21:1539–58. doi: 10.1002/sim.v21:11, PMID: 12111919

[24] Egger M Davey Smith G Schneider M Minder C . Bias in meta-analysis detected by a simple, graphical test. BMJ (Clinical Res ed). (1997) 315:629–34. doi: 10.1136/bmj.315.7109.629, PMID: 9310563 PMC2127453

[25] Adamkiewicz M Bryniarski P Kowalik M Burzyński B Rajwa P Paradysz A . Lymphocyte-to-monocyte ratio is the independent prognostic marker of progression in patients undergoing BCG-immunotherapy for bladder cancer. Front Oncol. (2021) 11:655000. doi: 10.3389/fonc.2021.655000, PMID: 33842371 PMC8033152

[26] Albisinni S Moussa I Aoun F Quackels T Assenmacher G Peltier A . The impact of postoperative inflammatory biomarkers on oncologic outcomes of bladder cancer. Progres en urologie: J l’Association francaise d’urologie la Societe francaise d’urologie. (2019) 29:270–81. doi: 10.1016/j.purol.2019.02.008, PMID: 30954405

[27] Batur AF Aydogan MF Kilic O Korez MK Gul M Kaynar M . Comparison of De Ritis Ratio and other systemic inflammatory parameters for the prediction of prognosis of patients with transitional cell bladder cancer. Int J Clin practice. (2021) 75:e13743. doi: 10.1111/ijcp.13743, PMID: 32991771

[28] Cantiello F Russo GI Vartolomei MD Farhan ARA Terracciano D Musi G . Systemic inflammatory markers and oncologic outcomes in patients with high-risk non-muscle-invasive urothelial bladder cancer. Eur Urol Oncol. (2018) 1:403–10. doi: 10.1016/j.euo.2018.06.006, PMID: 31158079

[29] D’Andrea D Moschini M Gust KM Abufaraj M Özsoy M Mathieu R . Lymphocyte-to-monocyte ratio and neutrophil-to-lymphocyte ratio as biomarkers for predicting lymph node metastasis and survival in patients treated with radical cystectomy. J Surg Oncol. (2017) 115:455–61., PMID: 28105663 10.1002/jso.24521

[30] Karan C Yaren A Demirel BC Dogan T Ozdemir M Demiray AG . Pretreatment PLR is preferable to NLR and LMR as a predictor in locally advanced and metastatic bladder cancer. Cancer diagnosis prognosis. (2023) 3:706–15. doi: 10.21873/cdp.10275, PMID: 37927800 PMC10619568

[31] Kim K Yu J Park JY Baek S Hwang JH Choi WJ . Low preoperative lymphocyte-to-monocyte ratio is predictive of the 5-year recurrence of bladder tumor after transurethral resection. J personalized Med. (2021) 11. doi: 10.3390/jpm11100947, PMID: 34683088 PMC8540090

[32] Kool R Marcq G Shinde-Jadhav S Mansure JJ Saleh R Rajan R . Role of serum lymphocyte-derived biomarkers in nonmetastatic muscle-invasive bladder cancer patients treated with trimodal therapy. Eur Urol Open Sci. (2022) 36:26–33. doi: 10.1016/j.euros.2021.11.011, PMID: 35098169 PMC8783035

[33] Lee SM Russell A Hellawell G . Predictive value of pretreatment inflammation-based prognostic scores (neutrophil-to-lymphocyte ratio, platelet-to-lymphocyte ratio, and lymphocyte-to-monocyte ratio) for invasive bladder carcinoma. Korean J urology. (2015) 56:749–55. doi: 10.4111/kju.2015.56.11.749, PMID: 26568792 PMC4643170

[34] Li DX Wang XM Feng DC Zhang FC Wu RC Shi X . Lymphocyte-to-monocyte ratio (LMR) during induction is a better predictor than preoperative LMR in patients receiving intravesical bacillus calmette -guerin for non-muscle-invasive bladder cancer. Front Oncol. (2022) 12:937638. doi: 10.3389/fonc.2022.937638, PMID: 35903700 PMC9314647

[35] Shi H Wang K Yuan J Mao W Wu Z Liu Q . A high monocyte-to-lymphocyte ratio predicts poor prognosis in patients with radical cystectomy for bladder cancer. Trans Cancer Res. (2020) 9:5255–67. doi: 10.21037/tcr-20-1060, PMID: 35117892 PMC8798907

[36] Tang X Cao Y Liu J Wang S Yang Y Du P . Diagnostic and predictive values of inflammatory factors in pathology and survival of patients undergoing total cystectomy. Mediators inflammation. (2020) 2020:9234067. doi: 10.1155/2020/9234067, PMID: 33029106 PMC7532356

[37] Wang Q Huang T Ji J Wang H Guo C Sun X . Prognostic utility of the combination of pretreatment monocyte-to-lymphocyte ratio and neutrophil-to-lymphocyte ratio in patients with NMIBC after transurethral resection. Biomarkers Med. (2019) 13:1543–55. doi: 10.2217/bmm-2019-0398, PMID: 31621380

[38] Yıldız HA Değer MD Aslan G . Prognostic value of preoperative inflammation markers in non-muscle invasive bladder cancer. Int J Clin practice. (2021) 75:e14118., PMID: 33636055 10.1111/ijcp.14118

[39] Yoshida T Kinoshita H Yoshida K Mishima T Yanishi M Inui H . Prognostic impact of perioperative lymphocyte-monocyte ratio in patients with bladder cancer undergoing radical cystectomy. Tumour biology: J Int Soc Oncodevelopmental Biol Med. (2016) 37:10067–74. doi: 10.1007/s13277-016-4874-8, PMID: 26819209

[40] Zhang GM Zhu Y Luo L Wan FN Zhu YP Sun LJ . Preoperative lymphocyte-monocyte and platelet-lymphocyte ratios as predictors of overall survival in patients with bladder cancer undergoing radical cystectomy. Tumour biology: J Int Soc Oncodevelopmental Biol Med. (2015) 36:8537–43. doi: 10.1007/s13277-015-3613-x, PMID: 26032095

[41] Zhang X Liu Q Yi K Liu S Lan J . The prognostic value of the combination of the prognostic nutritional index and the lymphocyte: monocyte ratio for the prediction of patients with muscle-invasive bladder cancer. Archivos espanoles urologia. (2024) 77:164–72. doi: 10.56434/j.arch.esp.urol.20247702.22, PMID: 38583009

[42] Sylvester RJ Rodríguez O Hernández V Turturica D Bauerová L Bruins HM . European Association of Urology (EAU) prognostic factor risk groups for non–muscle-invasive bladder cancer (NMIBC) incorporating the WHO 2004/2016 and WHO 1973 classification systems for grade: an update from the EAU NMIBC Guidelines Panel. Eur urology. (2021) 79:480–8. doi: 10.1016/j.eururo.2020.12.033, PMID: 33419683

[43] Alqurashi YE . Lymphocyte-activation gene 3 (LAG-3) as a promising immune checkpoint in cancer immunotherapy: From biology to the clinic. Pathology Res practice. (2024) 254:155124. doi: 10.1016/j.prp.2024.155124, PMID: 38295462

[44] Tian W Wei W Qin G Bao X Tong X Zhou M . Lymphocyte homing and recirculation with tumor tertiary lymphoid structure formation: predictions for successful cancer immunotherapy. Front Immunol. (2024) 15:1403578. doi: 10.3389/fimmu.2024.1403578, PMID: 39076974 PMC11284035

[45] Wu Y Yuan M Wang C Chen Y Zhang Y Zhang J . T lymphocyte cell: A pivotal player in lung cancer. Front Immunol. (2023) 14:1102778. doi: 10.3389/fimmu.2023.1102778, PMID: 36776832 PMC9911803

[46] Kannen V Grant DM Matthews J . The mast cell-T lymphocyte axis impacts cancer: Friend or foe? Cancer Lett. (2024) 588:216805. 38462035 10.1016/j.canlet.2024.216805

[47] Li YQ Chen XM Si GF Yuan XM . Progress of lymphocyte activation gene 3 and programmed cell death protein 1 antibodies for cancer treatment: A review. Biomolecules biomedicine. (2024) 24:764–74. doi: 10.17305/bb.2024.10339, PMID: 38581716 PMC11293232

[48] Hu W Bian Y Ji H . TIL therapy in lung cancer: current progress and perspectives. Advanced Sci. (2024):e2409356. doi: 10.1002/advs.202409356, PMID: 39422665 PMC11633538

[49] Jiang J Shu W Yao Q . Research advances on TIL therapy for colorectal cancer. Clin Trans Oncol Off Publ Fed Spanish Oncol Societies Natl Cancer Institute Mexico. (2024) 26:2917–23. doi: 10.1007/s12094-024-03530-4, PMID: 38806995

[50] Lok V Olson-McPeek S Spiegelhoff G Cortez J Detz D Czerniecki B . Immunotherapies in breast cancer: harnessing the cancer immunity cycle. Expert Opin Ther Targets. (2024), 1–11. doi: 10.1080/14728222.2024.2427038, PMID: 39523444

[51] Plaugher DR Childress AR Gosser CM Esoe DP Naughton KJ Hao Z . Therapeutic potential of tumor-infiltrating lymphocytes in non-small cell lung cancer. Cancer letters. (2024) 605:217281. doi: 10.1016/j.canlet.2024.217281, PMID: 39369769 PMC11560632

[52] Ammarah U Pereira-Nunes A Delfini M Mazzone M . From monocyte-derived macrophages to resident macrophages-how metabolism leads their way in cancer. Mol Oncol. (2024) 18:1739–58. doi: 10.1002/1878-0261.13618, PMID: 38411356 PMC11223613

[53] Yoshimura T Li C Wang Y Matsukawa A . The chemokine monocyte chemoattractant protein-1/CCL2 is a promoter of breast cancer metastasis. Cell Mol Immunol. (2023) 20:714–38. doi: 10.1038/s41423-023-01013-0, PMID: 37208442 PMC10310763

[54] Fendl B Berghoff AS Preusser M Maier B . Macrophage and monocyte subsets as new therapeutic targets in cancer immunotherapy. ESMO Open. (2023) 8:100776. doi: 10.1016/j.esmoop.2022.100776, PMID: 36731326 PMC10024158

[55] Amer HT Stein U El Tayebi HM . The monocyte, a maestro in the tumor microenvironment (TME) of breast cancer. Cancers (Basel). (2022) 14. doi: 10.3390/cancers14215460, PMID: 36358879 PMC9658645

[56] Kawai M Hirono S Okada K-I Miyazawa M Shimizu A Kitahata Y . Low lymphocyte monocyte ratio after neoadjuvant therapy predicts poor survival after pancreatectomy in patients with borderline resectable pancreatic cancer. Surgery. (2019) 165:1151–60. doi: 10.1016/j.surg.2018.12.015, PMID: 30765142

[57] Poirier A Tremblay ML . Pharmacological potentiation of monocyte-derived dendritic cell cancer immunotherapy. Cancer immunology immunotherapy: CII. (2023) 72:1343–53. doi: 10.1007/s00262-022-03333-y, PMID: 36441193 PMC10992185

[58] Ino Y Yamazaki-Itoh R Shimada K Iwasaki M Kosuge T Kanai Y . Immune cell infiltration as an indicator of the immune microenvironment of pancreatic cancer. Br J cancer. (2013) 108:914–23. doi: 10.1038/bjc.2013.32, PMID: 23385730 PMC3590668

