# Supplementary material for: Prognostic value of lymphocyte to monocyte ratio for the patients with bladder cancer: a systematic review and meta-analysis
Source: Front Oncol. 2025 Oct 3;15:1601040. doi: 10.3389/fonc.2025.1601040 (PMC12532007; doi:10.3389/fonc.2025.1601040)
Supplement: Supplementary file 1 [file Table1.docx]

**TableS1** Literature search strategy

Pubmed-69

(((("Lymphocytes"[Mesh]) OR (((Lymphocyte) OR (Lymphoid Cells)) OR (Lymphoid Cell))) AND (("Monocytes"[Mesh]) OR (Monocyte))) AND (ratio)) AND (("Urinary Bladder Neoplasms"[Mesh]) OR ((((((((((Urinary Bladder Neoplasm) OR (Bladder Neoplasms)) OR (Bladder Neoplasm)) OR (Bladder Tumors)) OR (Bladder Tumor)) OR (Urinary Bladder Cancer)) OR (Bladder Cancer)) OR (Bladder Cancers)) OR (Cancer of Bladder)) OR (Cancer of the Bladder)))

Embase-117


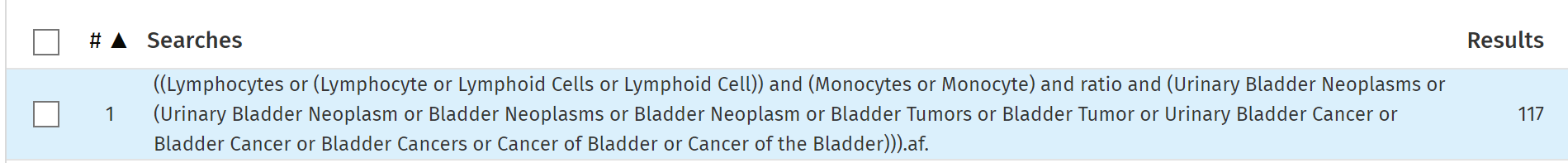


Cochrane-1


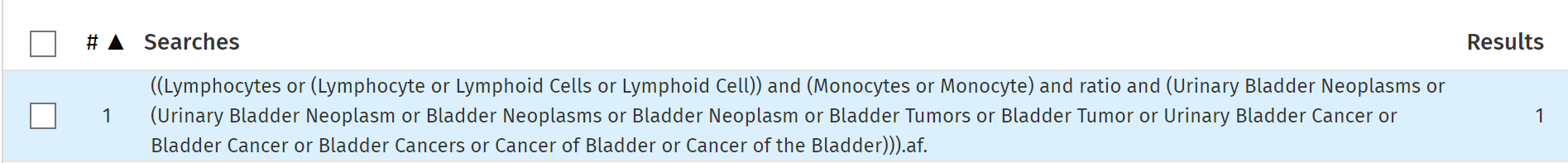


WOS-88

((((Lymphocytes) OR (((Lymphocyte) OR (Lymphoid Cells)) OR (Lymphoid Cell))) AND ((Monocytes) OR (Monocyte))) AND (ratio)) AND ((Urinary Bladder Neoplasms) OR ((((((((((Urinary Bladder Neoplasm) OR (Bladder Neoplasms)) OR (Bladder Neoplasm)) OR (Bladder Tumors)) OR (Bladder Tumor)) OR (Urinary Bladder Cancer)) OR (Bladder Cancer)) OR (Bladder Cancers)) OR (Cancer of Bladder)) OR (Cancer of the Bladder))) (Topic)
